# Supplementary material for: Workplace bullying, psychological hardiness, and accidents and injuries in nursing: A moderated mediation model
Source: PLoS One. 2021 Jan 8;16(1):e0244426. doi: 10.1371/journal.pone.0244426 (PMC7793278; doi:10.1371/journal.pone.0244426)
Supplement: S1 File — (DOC) [file pone.0244426.s001.doc]

S1 Appendix

Study 1 Measurement properties

| **Construct** | **Abbr.** | **Item statements** | **Factor loading** | **CR** | **AVE** |
| --- | --- | --- | --- | --- | --- |
| **Workplace bullying** | NA1 | Someone withholding necessary information so that your work gets complicated | 0.78 | 0.968 | 0.772 |
|  | NA2 | Gossip or rumors about you | 0.87 |  |  |
|  | NA3 | Social exclusion from co-workers or work group activities | 0.81 |  |  |
|  | NA4 | Repeated offensive remarks about you or your private life | 0.89 |  |  |
|  | NA5 | Insults | 0.93 |  |  |
|  | NA6 | Repeated reminders about your blunders or mistakes | 0.93 |  |  |
|  | NA7 | Silence or hostility as a response to your questions or attempts at conversations | 0.92 |  |  |
|  | NA8 | Devaluing of your work and efforts | 0.90 |  |  |
|  | NA9 | So called ‘funny surprises’ | 0.86 |  |  |
| **Psychological stress** | STRES1 | Did you feel tired out for no good reason? | 0.69 | 0.964 | 0.729 |
|  | STRES2 | Did you feel nervous? | 0.81 |  |  |
|  | STRES3 | Did you feel so nervous that nothing could calm you down? | 0.87 |  |  |
|  | STRES4 | Did you feel hopeless? | 0.90 |  |  |
|  | STRES5 | Did you feel restless or fidgety? | 0.89 |  |  |
|  | STRES6 | Did you feel so restless that you could not sit still? | 0.87 |  |  |
|  | STRES7 | Did you feel depressed? | 0.88 |  |  |
|  | STRES8 | Did you feel that everything was an effort? | 0.84 |  |  |
|  | STRES9 | Did you feel so sad that nothing could cheer you up? | 0.88 |  |  |
|  | STRES10 | Did you feel worthless? | 0.89 |  |  |
| **Workplace accidents and injuries** | BN1 | Work-related stress from occupational violence, bullying/harassment, workplace conflicts and work pressure | 0.76 | 0.944 | 0.773 |
|  | BN2 | Work-related accidents and or injuries from manual handling | 0.89 |  |  |
|  | BN3 | Work-related accidents and or injuries from exposure to hazardous substances | 0.93 |  |  |
|  | BN4 | Work-related accidents and or injuries from “trips, slips and falls” | 0.94 |  |  |
|  | BN5 | Work-related accidents and or injuries from fatigue (particularly associated with shift work, long hours or being 'on the move' the whole time) | 0.86 |  |  |

S2 Appendix

Study 2 Measurement properties

| **Construct** | **Abbr.** | **Item statements** | **Factor loading** | **CR** | **AVE** |
| --- | --- | --- | --- | --- | --- |
| **Workplace bullying** | NA1 | Someone withholding necessary information so that your work gets complicated | 0.79 | 0.942 | 0.644 |
|  | NA2 | Gossip or rumors about you | 0.82 |  |  |
|  | NA3 | Social exclusion from co-workers or work group activities | 0.74 |  |  |
|  | NA4 | Repeated offensive remarks about you or your private life | 0.81 |  |  |
|  | NA5 | Insults | 0.82 |  |  |
|  | NA6 | Repeated reminders about your blunders or mistakes | 0.81 |  |  |
|  | NA7 | Silence or hostility as a response to your questions or attempts at conversations | 0.84 |  |  |
|  | NA8 | Devaluing of your work and efforts | 0.83 |  |  |
|  | NA9 | So called ‘funny surprises’ | 0.77 |  |  |
| **Psychological stress** | STRES1 | Did you feel tired out for no good reason? | Removed due to low factor loading | 0.914 | 0.543 |
|  | STRES2 | Did you feel nervous? | 0.67 |  |  |
|  | STRES3 | Did you feel so nervous that nothing could calm you down? | 0.77 |  |  |
|  | STRES4 | Did you feel hopeless? | 0.82 |  |  |
|  | STRES5 | Did you feel restless or fidgety? | 0.67 |  |  |
|  | STRES6 | Did you feel so restless that you could not sit still? | 0.71 |  |  |
|  | STRES7 | Did you feel depressed? | 0.74 |  |  |
|  | STRES8 | Did you feel that everything was an effort? | 0.75 |  |  |
|  | STRES9 | Did you feel so sad that nothing could cheer you up? | 0.78 |  |  |
|  | STRES10 | Did you feel worthless? | 0.71 |  |  |
| **Workplace accidents and injuries** | WA1 | Fractures | 0.90 | 0.961 | 0.754 |
|  | WA2 | Dislocations, sprains, and strains | 0.79 |  |  |
|  | WA3 | Bruising and crushing | 0.83 |  |  |
|  | WA4 | Superficial wounds (i.e., scratches and abrasions) | 0.73 |  |  |
|  | WA5 | Open wounds (i.e., cuts, lacerations, and punctures) | 0.89 |  |  |
|  | WA6 | Burns and scalds | 0.94 |  |  |
|  | WA7 | Eye injuries | 0.89 |  |  |
|  | WA8 | Concussions and other head injuries | 0.95 |  |  |
| **Psychological hardiness** | PH1 | Despite setbacks, I remain committed to accomplishing job tasks | 0.70 | 0.852 | 0.501 |
|  | PH2 | When necessary I am willing to work extra hard | 0.71 |  |  |
|  | PH3 | When a problem occurs at work, I am usually able to deal with it | 0.77 |  |  |
|  | PH4 | I am in control of most things that happen to me at work | 0.62 |  |  |
|  | PH5 | I enjoy facing new challenges at work | 0.68 |  |  |
|  | PH6 | I am able to cope with unexpected problems at work | 0.72 |  |  |
