# Supplementary material for: Workplace bullying, psychological hardiness, and accidents and injuries in nursing: A moderated mediation model
Source: PLoS One. 2021 Jan 8;16(1):e0244426. doi: 10.1371/journal.pone.0244426 (PMC7793278; doi:10.1371/journal.pone.0244426)
Supplement: Attachment — Submitted filename: PONE D 20 12891 reviewers comments.doc [file pone.0244426.s002.doc]

Dear Editor

Thank you for your comments. We have revised our manuscript and have revised the manuscript according to your request and the comments made by the reviewers.

| **Most recent comments from the journal’s editorial office (03 Nov 2020):** \| **Actions undertaken**; 1.) Please confirm whether the data located in your manuscript constitutes the minimal data set. The minimal data set is defined as the data set used to reach the conclusions drawn in the manuscript with related metadata and methods, and any additional data required to replicate the reported study findings in their entirety. This may include: a.) The values behind the means, standard deviations and other measures reported; b.) The values used to build graphs; c.) The points extracted from images for analysis (https://journals.plos.org/plosone/s/data-availability#loc-minimal-data-set-definition). \| We confirm the data located in your manuscript constitutes the minimal data set. This may include: a.) The values behind the means, standard deviations and other measures reported; b.) The values used to build graphs; c.) The points extracted from images for analysis (https://journals.plos.org/plosone/s/data-availability#loc-minimal-data-set-definition).; 2.) If the minimal data set is located in your manuscript, please confirm whether we may update your Data Availability Statement as follows: \| Please update our data availability statement with: “All relevant data are within the manuscript.” This is attached as supplementary files linked to this article.; 3.) If additional data is required to fulfill the minimal data set, please either upload it as Supporting Information or to a stable public repository. Please also provide the relevant URLs, DOIs, or accession numbers for any data uploaded to a repository. For a list of acceptable repositories, please see http://journals.plos.org/plosone/s/data-availability#loc-recommended-repositories. \| Thank you. We have provided two supplementary documents attached to this manuscript to comply the minimum data requirement.; 2. Thank you for including your ethics statement on the online submission form: "Auckland University of Technology Ethics Committee Approval number: Lamm 15373 Written consent obtained from the ethics committee.". * To help ensure that the wording of your manuscript is suitable for publication, would you please also add this statement at the beginning of the Methods section of your manuscript file to include the full name of the ethics committee approving your study. \| We have added the ethics statement at the beginning of the Methods section of our manuscript, as per your request. | | |
| --- | --- | --- |
| **Editor’s comments from Revision decision #1** | | **Actions undertaken** |
| 1. Please ensure that your manuscript meets PLOS ONE's style requirements, including those for file naming. The PLOS ONE style templates can be found at  https://journals.plos.org/plosone/s/file?id=wjVg/PLOSOne_formatting_sample_main_body.pdf and  https://journals.plos.org/plosone/s/file?id=ba62/PLOSOne_formatting_sample_title_authors_affiliations.pdf | | Thank you for your advice. We have restructure the revised manuscript in accordance to the style template. |
| 2. Thank you for stating the following financial disclosure: 'The funders had no role in study design, data collection and analysis, decision to publish, or preparation of the manuscript.'  At this time, please address the following queries:  a) Please clarify the sources of funding (financial or material support) for your study. List the grants or organizations that supported your study, including funding received from your institution.  b) State what role the funders took in the study. If the funders had no role in your study, please state: “The funders had no role in study design, data collection and analysis, decision to publish, or preparation of the manuscript.”  c) If any authors received a salary from any of your funders, please state which authors and which funders.  d) If you did not receive any funding for this study, please state: “The authors received no specific funding for this work.”  Please include your amended statements within your cover letter; we will change the online submission form on your behalf. | | Thank you for your advice.   a) Funding was provided by the Auckland University of Technology as a research grant awarded to Teo, Lamm and Boocock. b) The funder had no role in study design, data collection and analysis, decision to publish, or preparation of the manuscript. c) Authors Trevelyan, Lamm, and Boocock are full time employees of the funder (Auckland University of Technology).   d) N/A |
| 3. In your Data Availability statement, you have not specified where the minimal data set underlying the results described in your manuscript can be found. PLOS defines a study's minimal data set as the underlying data used to reach the conclusions drawn in the manuscript and any additional data required to replicate the reported study findings in their entirety. All PLOS journals require that the minimal data set be made fully available. For more information about our data policy, please see http://journals.plos.org/plosone/s/data-availability. | | We were unable to share the **raw data** as this is prohibited by the institutional ethics review board. The data reported in this paper is part of a larger study.  However, we have provided the minimum dataset require as per PLOS One’s instruction on the journal’s website (https://journals.plos.org/plosone/s/data-availability) where “authors should submit the following data:  1. The values behind the means, standard deviations and other measures reported – this is in the descriptive statistics table (see Table 2 and Table 4);  2. The values used to build graphs (see Figure );  3. The points extracted from images for analysis (not applicable).  Authors do not need to submit their entire data set if only a portion of the data was used in the reported study. Also, authors do not need to submit the raw data collected during an investigation if the standard in the field is to share data that have been processed. |
| **Comments: Reviewer #1** | Actions undertaken | |
| It is a very well written manuscript. You have studied a really important field and you reached some valuable conclusions that research community can gain from that. Methodology and results are appropriately described. | Thank you for your support and comments about the contribution of our paper. | |
| **Reviewer #2 Comments** |  | |
| 1. Please update reference Fevre and colleagues (2011) and information about the UK. | We have updated the reference according to this journal’s preferred referencing style.  Fevre et al (2011) and the UK context was correctly referenced in the original manuscript (please refer to page 3). We can confirm that the original reference used in the manuscript was correct (see https://www.researchgate.net/publication/258206731_Insight_into_ill-treatment_patterns_causes_and_solutions/link/57fb640608ae8da3ce60d58d/download). | |
| 2. Please write research ethics: ethical code, getting Satisfaction. | This was reported in the manuscript portal: Auckland University of Technology Ethics Committee (number: Lamm 15373). | |
| 3. Please add time of research. | The research was undertaken over 2015 and 2016 as there were two studies in the paper (see p. 10 and p.12). | |
| 4. Please describe research limitations. | Research limitation was outlined in the original manuscript and it was signed post on page 16-17. | |
| 5. I think type of wards in which the nurses studied worked is important. Can be added | This is a great idea. As the study is based on a cross sectional dataset, we did not collect any information on the ward of the participants. This is now included as a suggestion for future study. Please see the revised section on page 16-17. | |
| 6. Manuscript is too long | We have shortened the manuscript and the number of references. Its current length is 4670 words (excluding references, tables, and figures). | |
